# Butyrate ameliorates colorectal cancer through regulating intestinal microecological disorders

**DOI:** 10.1097/CAD.0000000000001413

**Published:** 2022-10-24

**Authors:** Jingjing Kang, Mingzhong Sun, Yi Chang, Hongmei Chen, Juan Zhang, Xiaodong Liang, Tengfei Xiao

**Affiliations:** a Department of Clinical Laboratory, Yancheng First Hospital, Affiliated Hospital of Nanjing University Medical School, The First people’s Hospital of Yancheng; b Department of Clinical Laboratory; c Department of Pathology, The Sixth Affiliated Hospital of Nantong University, Yancheng Third People’s Hospital, Yancheng, Jiangsu, China

**Keywords:** 16S rDNA, butyrate, colorectal cancer, tumor microbiota

## Abstract

The occurrence and progression of colorectal cancer (CRC) are closely related to intestinal microecological disorders. Butyrate, the representative of short chain fatty acids, possess anti-inflammatory and antioxidant effects, and its antitumor effect has been gradually paid attention to. In this study, azoxymethane/dextran sodium sulfate induced mouse CRC model was used to explore the role and mechanism of butyrate in regulating colon cancer and its intestinal microecological balance. Outcomes exhibited that butyrate alleviated weight loss, disease activity index, and survival in CRC mice and inhibited tumor number and progression. Further research revealed that butyrate restrained the aggregation of harmful while promoting the colonization of beneficial flora, such as *Actinobacteriota, Bifidobacteriales* and *Muribaculacea* through 16S rDNA sequence analysis. This study confirmed that butyrate can ameliorate CRC by repairing intestinal microecology, providing ideas and evidence for chemical prophylactic agents, such as butyrate to remedy tumors and regulate tumor microbiota.

## Introduction

Colorectal cancer (CRC) represents a substantial burden for the public healthcare system and patients, and its incidence remains stubbornly high [1]. Its mortality has been soothing in recent years because of the improvement of modern gastrointestinal examination technology, the strengthening of patients’ health care awareness and the promotion of treatment strategies year by year [2]. The mechanism of CRC is complex and multifactorial, which involves excessive immune activation, individual genetics, organismal drug history and inappropriate lifestyle [3–5]. With the development of sequencing technology, intestinal microecological disorder, which can trigger inflammatory cascade, deserves to increase attention in CRC [6]. It can be traced back to 1975, the investigator stated that the probability of CRC formation in sterile rats induced by carcinogens was lower than that in normal rats [7]. Research has further proved that transplantation of intestinal flora from CRC mice will aggravate the occurrence and development of intestinal polyps [8].

Butyrate is a kind of short-chain fatty acid (SCFA), which can be synthesized by a variety of Gram-positive bacteria, typically *Faecalibacterium* and *Roseburia* [9]. Butyrate can be applied as the mainstay of epithelial cell energy supply after undergoing oxidation and tricarboxylic acid cycle in the colon [10,11]. In most cases, butyrate plays a heroic role in CRC, such as downregulating IL-6, enhancing the antibacterial ability of macrophages and exerting the positive energy of protecting CRC from deterioration [12–14]. Relevant in-vitro studies demonstrated that butyrate suppresses histone deacetylase, then terminates the cell cycle in the G1 phase and strengthens cell differentiation simultaneously, such effect is indispensable for tumor containment [15,16]. In the CRC model, butyrate combined with its specific receptor GPR109A stimulated the differentiation of T cells expressing IL-10 and induced the apoptosis of internal epithelial cells (IECs) [17,18]; Limiting the proliferation of IEC by mediating the transcription factor FOXO3 is also a form of butyrate to curb cancer [19,20]. Butyrate regulates inflammation and promotes the integrity of intestinal epithelial cells, but the internal study of butyrate in CRC is relatively vague, and its mechanism continues to be verified.

In the disrupted intestinal microecological world in CRC, the structure of harmonious symbiotic flora alters, and the original cooperative relationship of joint resistance to external harmful factors collapses [21,22]. Researchers confirmed that in CRC, the integrity of the colorectal barrier was impaired, contributing to bacterial translocations previously targeted at other sites and resulting in the aggregation and reproduction of invasive bacteria, and jointly promoting the production of inflammatory factors, such as IL-17A, IL-6, IL-27, etc [23,24]. This uncontrolled outbreak of inflammation will organize new microecology and promote the dominant growth of harmful flora [25]. Harmful flora will trigger toxic reactions, such as Escherichia coli activating polyketide synthase to release enterotoxin, incurring DNA damage and inflammation oxidative stress cascade, eventually formulating a vicious cycle [26–28]. Under the circumstance of CRC, the magnitude of butyrate-producing species decreased sharply, rendering the pathological injury of intestinal tumors more serious [29,30]. The research on butyrate and microbiota in CRC is infrequent. Therefore, this study aims to describe the occurrence and development of CRC and the change characteristics of intestinal microecological with butyrate treatment, so as to provide new evidence for CRC clinical prevention and treatment.

## Materials and methods

### Establishment of azoxymethane/dextran sodium sulfate induced CAC model

**Fig. 1:**
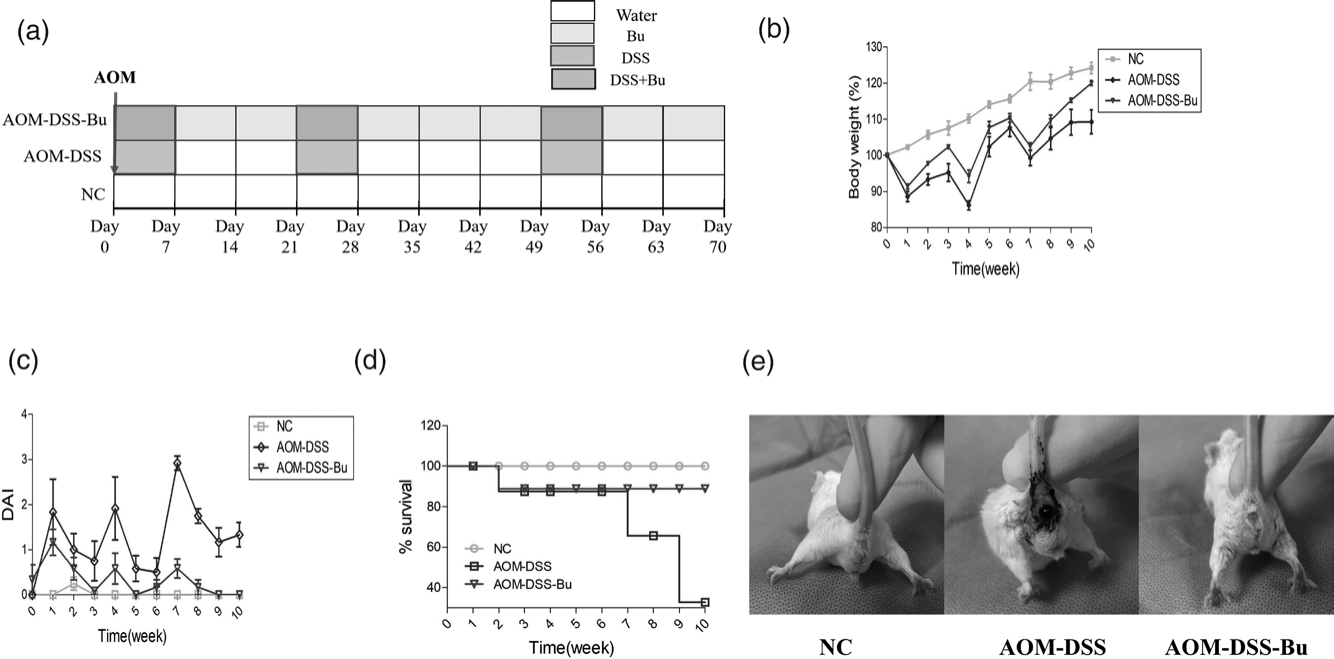
Butyrate relieves symptoms of AOM/DSS induced CAC. (a) Protocol of AOM/DSS induced CAC. (b) The body weight loss (*n* = 10 per group). (c) The disease activity index (DAI) (*n* = 10 per group). (d) Survival curve analysis (*n* = 10 per group). (e) The rectal prolapse of mice at the 10th week. Data are represented as the mean ± standard. *P < 0.05. AOM/DSS, azoxymethane-dextran sodium sulfate; CAC, colitis-associated colorectal cancer.

Balb/c male mice (*n* = 30) weighing about 20–22 g for 6–8 weeks were randomly divided into three groups, purchased from specific pathogen Free (Beijing) Biotechnology Co., Ltd. All animal experiments processes were approved by the Ethics Committee of Jiangsu Vocational College of Medicine (Yancheng, China), and abide by experimental animal operation specifications strictly. Three different treatment mice models, receiving sterilized water, grain, PBS and drugs, were carried out: negative control group (NC), azoxymethane-dextran sodium sulfate (AOM-DSS) model group and AOM-DSS-Bu group. After 1 week of preconditioning, the NC group was intraperitoneally injected with PBS, and AOM-DSS and AOM-DSS-Bu groups were intraperitoneally injected with 10 mg/kg AOM (Sigma Chemical Co. USA). The AOM-DSS and AOM-DSS-Bu groups were treated with water containing 2.5% DSS (2.5%, MP Biomedicals, Canada, M.W. = 36000–50000 kDa) for 7 days and followed with normal drinking water for 14 days. Taking the mentioned program for three cycles to establish the mouse colitis-associated colorectal cancer (CAC) model. The NC group drank normal water during the whole process whereas the AOM-DSS-Bu groups were gavaged with 2% butyrate (Sigma Chemical Co. USA) (Fig. 1a). All mice were weighed daily and examined for fecal traits, and analyzed for weight loss as well as disease activity index (DAI) (Table 1) [31]. The day of drinking DSS water was set as day 0, and all mice were killed on day 65 to obtain samples. Colon and spleen tissues of the mouse (4 μm thick) which formalin-fixed paraffin-embedded were stained by hematoxylin-eosin.

**Table 1:** Scoring standards of disease activity index

Scoring standards	0	1	2	3	4
Weight loss(%)	None	1–5	6–10	11–20	>20
Hemorrhage	None	FOBT+	Bloody stool	Bloody anus	Obvious
Stool property	Normal	Semi forming	Mushy	Anal paste	Liquid

FOBT, fecal occult blood test.

### Gut microbiome profiling and analysis

The aseptic operation was performed strictly to collect intestinal content which immediately froze at −80 ℃ for subsequent experiments. 16S rDNA amplicon sequencing and Illumina MiSeq were conducted at GENEWIZ. In brief, sample genomic DNA was extracted and qualified for the amplification and sequencing library construction of the 16S rDNA variable region by using a MetaVx Library Preparation kit (GENEWIZ) and then implementing high-throughput sequencing [32]. Bioinformation analysis first filters the original data, obtains effective sequences and then conducts cluster analysis to acquire operational taxonomic units (OTU) of different species to obtain species distribution information of each sample. Based on OTU analysis results, species richness and evenness of each group gained by multiple α diversity index analysis and depended on taxonomic information, community structure statistical analysis carried out at various taxonomic levels. The differences in community structure between different groups are intuitively displayed via the unweighted pair group method with arithmetic mean (UPGMA) cluster tree, principal co-ordinates analysis (PCoA), principal co-ordinates analysis (PCA), and so on.

### Statistical analysis

Data analysis was conducted via one-way analysis of variance (ANOVA) and presented as mean ± SEM. *P* values of < 0.05 is regarded as significant.

## Results

### Butyrate relieves symptoms of azoxymethane/dextran sodium sulfate induced CAC

The mice were divided into groups as shown in Fig. 1a and analyzed with their body weight, DAI and survival curve. In contrast to the steady rise in the NC group, the body weight of the AOM-DSS and AOM-DSS-Bu mice gradually rebounded after a sharp decrease during the week using DSS at the beginning of each cycle. Interestingly, the magnitude of body weight loss was blunted in CRC mice with butyrate treatment (Fig. 1b). A similar phenomenon occurs in DAI. Compared with the large rise and fall DAI index in the AOM-DSS group, the curve was lower and more flat in the AOM-DSS-Bu group (Fig. 1c). Meanwhile, with drinking butyrate, the survival rate of CRC mice was significantly improved (Fig. 1d), intestinal bleeding was significantly ameliorated, and their state was no longer flagging (Fig. 1e). To sum up, butyrate can be able to relieve general symptoms of AOM/DSS induced CAC.

### Butyrate attenuated the occurrence and progression of colorectal cancer

**Fig. 2:**
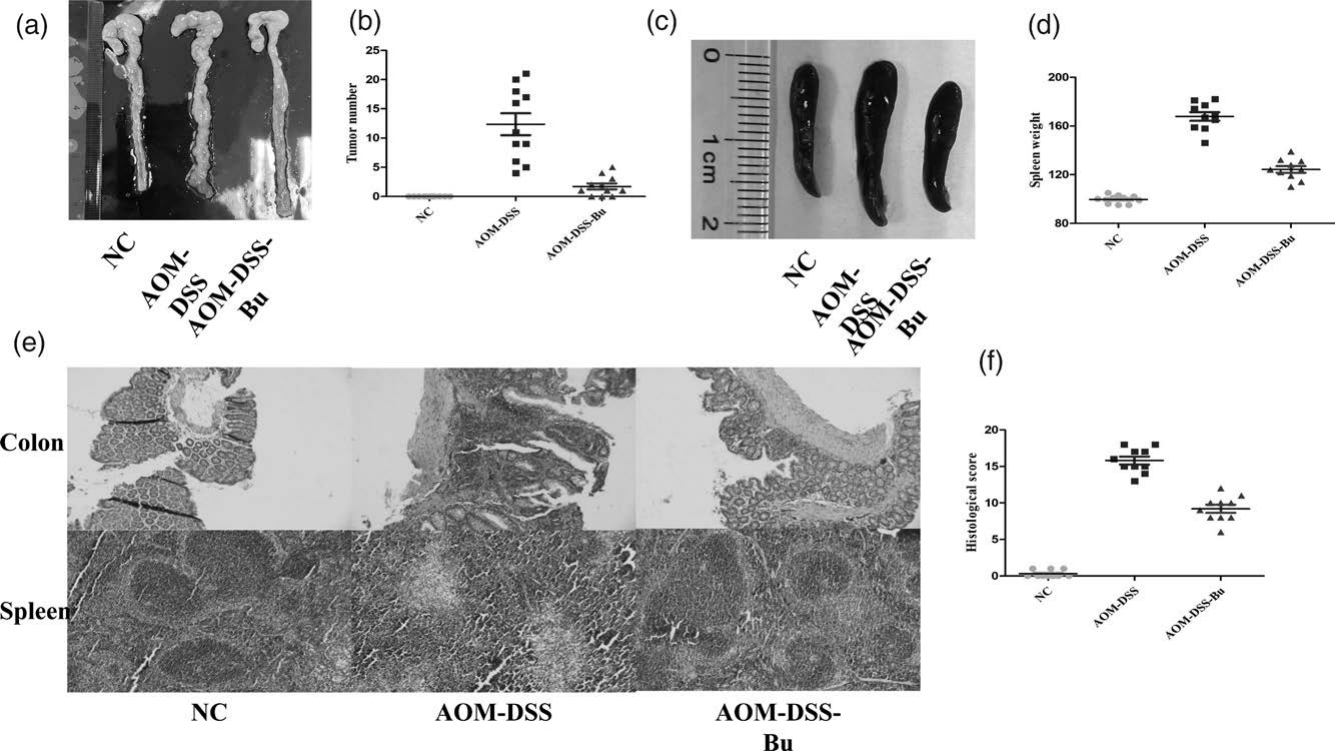
Butyrate attenuated the occurrence and progression of CRC. (a) Macroscopic morphology of colon tumors. (b) The number of colon tumors (*n* = 10 per group). (c) Macroscopic morphology of spleen. (d) The weight of spleen (*n* = 10 per group). (e) Hematoxylin-eosin (H&E) staining of colon and spleen (microscope ×40). (f) Histological score (*n* = 10 per group). Data are represented as the mean ± standard. *P < 0.05. CRC, colorectal cancer.

Once the induction of CRC was successful, the colon intestinal wall of CRC mice was thickened, the texture was stiffened and the intestinal loop was dilated. In the meantime, multiple obstructions and distortion along with multiple irregular neoplasias of the intestinal wall exacerbate the progression of colon cancer. Bleeding, erosions, ulceration damage severe in like manner. Butyrate reversed the bad situation, the colon length of CRC mice became longer, the thickening of the intestinal wall disappeared, and the degree of injury, bleeding and inflammation was significantly relieved, which attribute to butyrate treatment (Fig. 2a). Encouragingly, the number of intestinal tumors in the AOM-DSS-Bu group mice decreased sharply (Fig. 2b). Compared with the AOM-DSS group, the length of the spleen in the AOM-DSS-Bu group was abbreviated (Fig. 2c), and the weight of spleen was reduced significantly (Fig. 2d). Hematoxylin-eosin histology showed that a large number of inflammatory cells infiltrated in the colon of CRC mice, the gland structure collapsed and a large number of vacuoles and atypical hyperplasia were formed. In contrast, in the AOM-DSS-Bu group, the structural disorder recovered, the inflammatory cell infiltration was slight, and the number and scope of atypical hyperplasia lessened distinctly. Correspondingly, butyrate can also reverse the structural disorder of spleen nodules in CRC mice (Fig. 2e). Histological score refers to Table 2 [33], and confirmed that butyrate administration reduced the extent of AOM/DSS induced CAC (Fig. 2f). Therefore, it can be verified that butyrate possessed the capacity of preventing the occurrence and attenuating the progression of CRC.

**Table 2:** Histopathological score of colon

Scoring standards	0	1	2	3	4
Inflammation severity	None	Slight	Moderate	Serious	
Inflammatory range	None	Mucosa	Mucosa and submucosa	Transmural	
crypt damage	None	Mucosa 1/3	Mucosa 2/3	Crypts disappear but epithelial cells appear	Crypt and epithelial cells disappeared

### Butyrate changed gut flora diversity in colorectal cancer mice

For quality filtering, we first selected representative three mice from the NC group, and four mice from groups AOM-DSS and AOM-DSS-Bu, respectively, to collect their feces for 16S rRNA sequencing. To reflect the specie number in the microbial community, the abundance and diversity of species were estimated by alpha diversity analysis with the ACE, Chao, Shannon and Simpson indices. ACE and Chao indices based on the quantity of OTUs to assess the intestinal microbiota species richness; and Shannon and Simpson indices reflect alpha diversity were quantified to describe the enteric canal biodiversity among different groups.

**Fig. 3:**
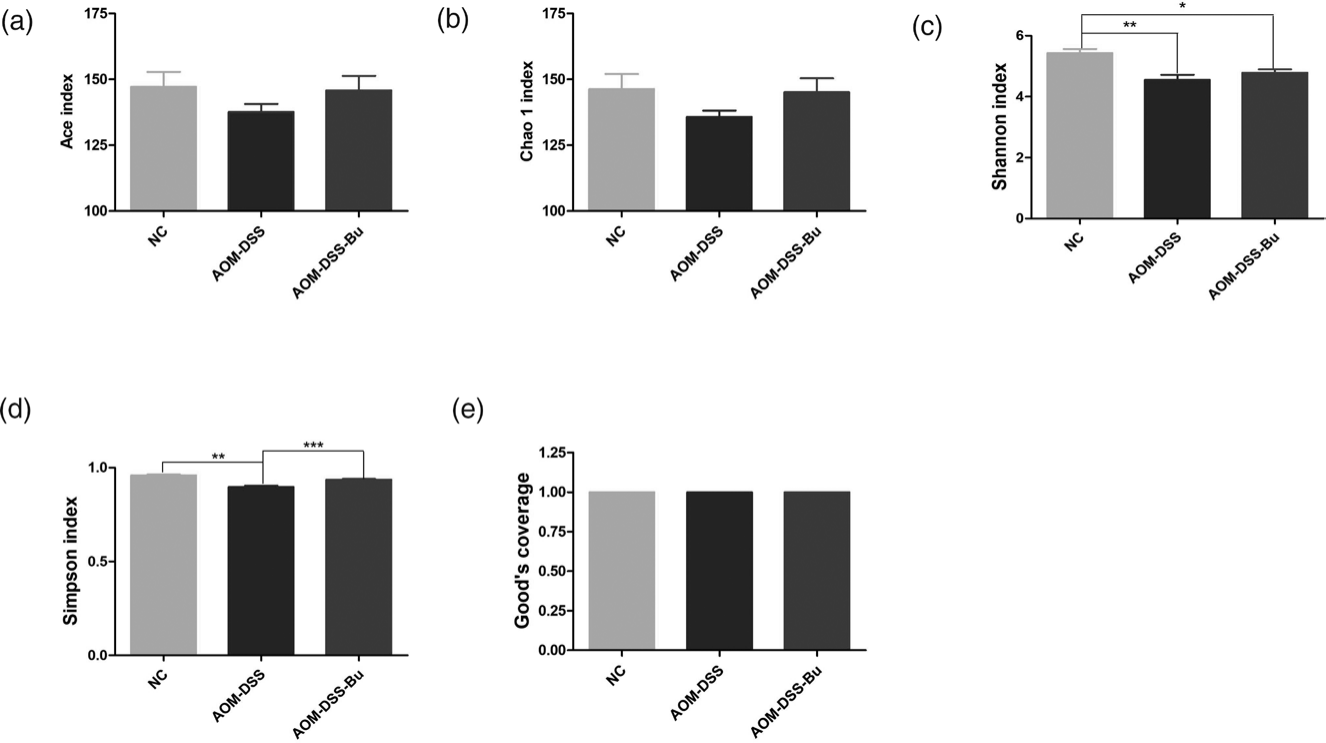
Butyrate changed gut flora diversity in CRC mice. (a) The ACE index. (b) The Chao 1 estimator. (c) The Shannon index. (d) The Simpson index. (e) Good’s coverage index. Data are represented as the mean ± standard. *P < 0.05, **P < 0.01 and ***P < 0.001. CRC, colorectal cancer.

No significant differences were observed on the basis of the result of ACE and Chao data analyses (Fig. 3a,b). The Shannon and Simpson indices in the CRC group were lower compared with those in the NC group, and the indices were firm up after butyrate treatment (Fig. 3c,d), illustrating that butyrate can alter the dramatic reduction of intestinal flora diversification triggered by CRC pathogenesis. Good’s coverage index indicated that 99.0% gut microbial taxa had been identified in mice with different treatments, indicating that the majority of microbial sequences measured in this research are representative (Fig. 3e). Above data indicate that CRC changes the abundance mildly as well as the diversity distinctly of mice gut microbes. And what makes sense is that butyrate consumption by CRC mice ameliorates their symptoms while also repairing the disrupted gut microecological diversity.

### Butyrate influenced on gut microbiota community composition in colorectal cancer mice

**Fig. 4:**
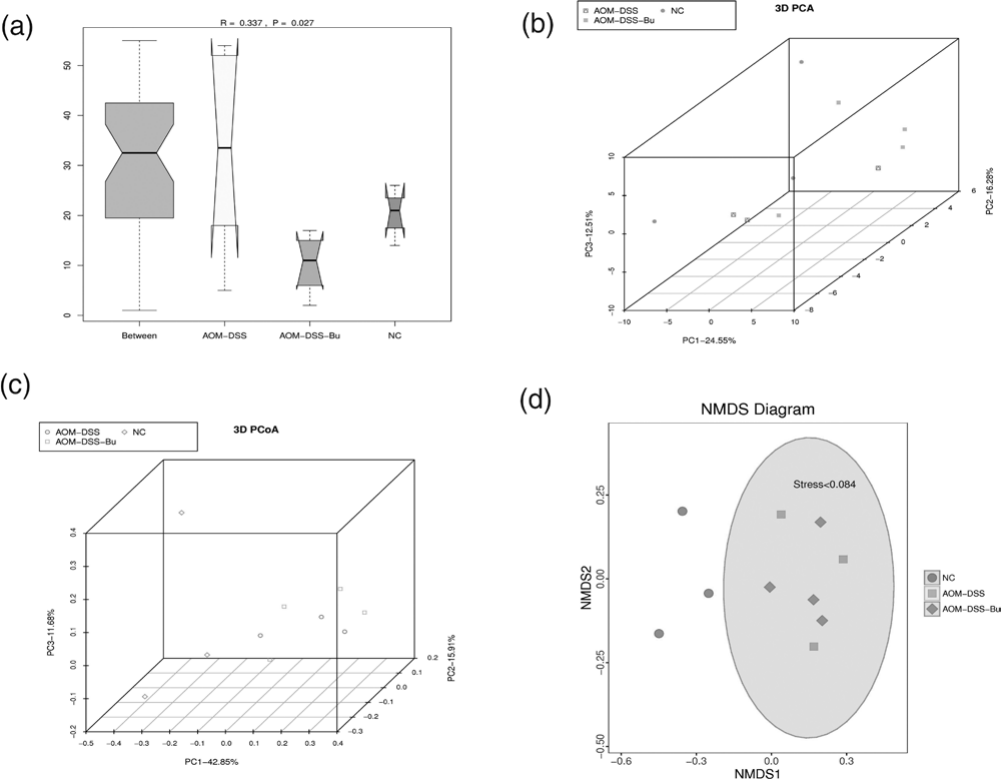
Butyrate influenced on gut microbiota community composition in CRC mice. (a) The ANOSIM index. (b) PCA of β diversity. (c) PCoA of β diversity. (d) NMDS analysis. ANOSIM, analysis of similarities; CRC, colorectal cancer; NMDS, Non-metric multidimensional scaling; PCA, principal co-ordinates analysis; PcoA, principal co-ordinates analysis.

To determine whether the intragroup differences in the study interfere with the intergroup differences, we used several beta diversity analyses such as the nonparametric test: analysis of similarities (ANOSIM), to analyze the inter sample distance matrix, so as to clarify the experimental significance (Fig. 4a); PCoA is a visualization method for data similarity or difference research (Fig. 4b) and the group variation analysis in PCA is reflected by the distance between points in the spatial positioning point map (Fig. 4c). Non-metric multidimensional scaling (NMDS) analysis employs variance decomposition to analyze the bacterial community distribution of each sample to reflect the similarity and difference (Fig. 4d).

We removed outliers before analysis. ANOSIM analysis explained that the bacterial microflora composition diversity of the three groups was significant (*P* = 0.027). The results from PCoA and PCA, NMDS showed that the gut microbiota composition of mice from groups AOM-DSS and AOM-DSS-Bu was significantly different from that of group NC, illustrating that CRC severely destroyed the harmonious and symbiotic microecological society, and reestablished an independent microecology. Thus, to some extent, butyrate influenced the CRC gut microbial community composition.

### Butyrate restored gut microecological dysbiosis in colorectal cancer mice

**Fig. 5:**
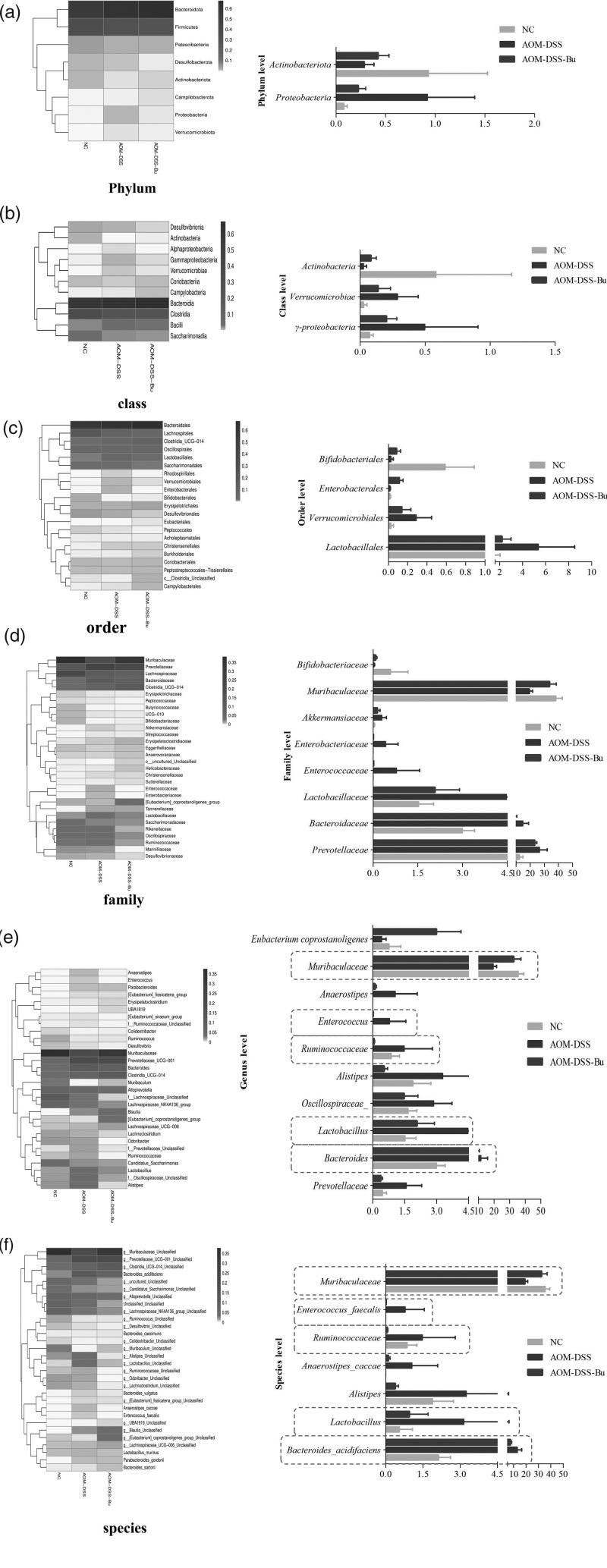
Butyrate restored gut microecological dysbiosis in CRC mice. Relative abundance analysis at (a) phylum level; (b) class level; (c) order level; (d) family level; (e) genus level; (f) species level among three groups. CRC, colorectal cancer.

At the phylum level, we found that *Bacteroidota* and *Firmicutes* accounted for a large proportion in the colon and the abundance of *Bacteroides* increased with the CRC construction compared with the NC group. The population of *Proteobacteria* elevated in the CRC group but which restrained along with the treatment of butyrate. *Actinobacteriota*, the main producer of antibiotics, can metabolize and generate a variety of antitumor, antibacterial and other high enzyme activities of biological resources [34]. In CRC mice induced by AOM/DSS, butyrate saved the abundance of *Actinobacteriota*, the dominant bacteria in the normal gut, from disappearing entirely (Fig. 5a).

At the class level, *Bacteroidia* and *Clostridia* are the principal colonization bacteria in the intestinal tract of each treatment group. Heat map data displayed that CRC contributes to the accumulation of *Bacilli* and the withering of resident flora such as *Saccharimonadia* compared with the NC group. The changes of the abundance of *Proteobacteria* and *Actinobacteria* in three groups were similar to that at the phylum level; and it should be noted that in group AOM-DSS, the number of *Verrucomicrobiae* increased significantly, but it was inhibited in group AOM-DSS-Bu (Fig. 5b).

At the order level, CRC not only stimulates the proportion of *Verrucomicrobiales* but also promotes the enrichment of *Lactobacillales* and *Enterobacterales* in the damaged intestine mucosa. Butyrate effectively prevented the population abundance of these colonies and facilitated the concentration of beneficial bacteria, such as *Clostridia* and *Eubacteriales. Bifidobacteriales*, key symbiotic bacteria colonized in the intestine, strengthen the intestinal barrier function and exert beneficial effects on inhibiting tumor and inflammation [35]. With the implementation of butyrate in the CRC group, the destroyed symbiosis and the population of *Bifidobacteriales* community rebounded (Fig. 5c).

At the family level, species classification and abundance differences are more diversified. In contrast to normal mice, The flora abundance of *Prevotellaceae, Bacteroidaceae, Lactobacillaceae, Enterococcaceae, Enterobacteriaceae and Akkermansiaceae* in the CRC group was considerably expanded. *Prevotellaceae* is known to produce enzymes that degrade mucin, which is committed to expanding intestinal wall permeability and even involved in increasing the risk of systemic autoimmunity [36]; *Enterococcaceae* and *Enterobacteriaceae* are certified as releasing enterotoxins with carcinogenic potential that cause DNA damage and activate genetic mutations [37]. *Akkermansiaceae* belongs to verrucomicrobia, which produces enzymes that degrade the intestinal mucus layer thereby worsening the intestinal barrier vulnerability [38]. Surprisingly, butyrate reduced the abundance of these harmful florae and promoted the abnormal intestinal microecological healthier. At the same time, the implementation of butyrate not only increased the number of *Bifidobacteriaceae* but also promoted the intestinal symbiotic flora such as *Eggerthellaceae, Clostridia UCG-014* and *Eubacterium coprostanoligenes* (Fig. 5d).

The fecal flora of mice in each group was richer and more varied in the genus and species level. In addition to the communities mentioned above, butyrate effectively controlled the stationing of *Oscillospiraceae, Alistipes* and *Anaerostipes*, which were significantly enriched in the intestine of CRC mice. When butyrate regulates the normalization of intestinal microecology in CRC mice, there is a flora: *Muribaculaceae*, butyrate promotes its recovery in CRC, which is worth our attention at family, genus and species level (Fig. 5e,f). *Muribaculaceae*, a gram-negative bacterium of the S24-7 family of Bacteroidetes, is a common host in the mouse gut and functionally differs from neighboring bacteriostases [39]. *Muribaculaceae* has been proven to contribute to the synthesis of SCFA and to be involved in mammalian longevity and antitumor careers [40].

Taken together, during the development of colorectal carcinogenesis, tumors destroy the health and constructed an independent and dangerous intestinal microecosystem, in which an enriched harmful flora community produces toxic substances, disrupts intestinal barrier function, exacerbates tumorigenesis and forms a vicious cycle. In the present study, butyrate was charged with the mission of reversing microbial dysbiosis, inhibiting the aggregation of harmful bacterial populations and reducing its damage to the intestinal wall barrier; and further devoted to strengthen the enrichment of beneficial flora, toughen intestinal defenses and reestablish a healthy and harmonious intestinal symbiotic environment.

## Discussion

CRC results from a combination including inflammatory cancer interactions, oncogenes and antioncogenes balance disturbances; and other than surgery, various biological agents targeting the prevention, treatment and prognosis improvement of CRC are more intensively investigated [41]. CRC is associated with a variety of gene mutations, such as kirsten rat sarcoma viral oncogene mutations, vrafmurine sarcoma viral oncegene homolog B mutations and high-degree CpG island methylator phenotype and so on [42]. Numerous studies have proved that the effect of chemotherapeutic drugs against CRC is related to the subtype of tumor mutation, as well as its cytotoxicity and short-term drug resistance, which lead to severe adverse consequences and limited curative effect [43]. A variety of natural biological agents have the functions of resisting pathogenic microorganisms and alleviating tumors. Resveratrol, a grape-derived stilbene, regulates the expression of PD-L1 and reduces the survival rate of colorectal tumor cells [44]; and ecteinascidin-743, isolated from sea squirt, has been revealed to improve the symptoms of patients with advanced CRC in phase I clinical trial [45]. Butyrate is a kind of SCFA with antibacterial and anti-inflammatory effects. Investigators elevated butyrate levels in the intestine, activating the transcription factor hypoxia inducible factor-1 in intestinal epithelial cells and thereby protecting against damage in *Clostridium difficile* induced colitis [46]. Another article demonstrated that butyrate preconditioning inhibited lipopolysaccharide-induced nuclear factor-k-gene binding P65 and protein kinase B (PKB) signaling pathways and successfully alleviated trinitrobenzene-sulfonic acid-induced colitis [47].

Butyrate was characterized for its antitumor effects in this study [10]. The antitumor effect of butyrate has been satisfactorily documented in different investigations. The effect of butyrate on intestinal cells is pleiotropic, being dependent on the dose and duration of action [48]. It plays a chemoprophylaxis role, promoting vigorous proliferation of colon epithelial cells and protecting the intestinal barrier by enhancing the repair ability of intestinal tight junction protein [49]. As expected, early supplementation with butyrate significantly alleviated weight loss, decreased the DAI index, improved survival and ameliorate the apathetic state of CRC mice. Further histological observation confirmed that butyrate functions to alleviate the degree of neoplastic lesions, lessen the number of tumor generation and repress the extent and scope of mucosal and glandular dysplasia in mice with colon cancer, demonstrating its ability to prevent and control CRC initiation and progression.

The human gut harbors a vast number of microbial populations, which are involved in immune response and metabolic activities [50]. In recent years, multiple research teams have focused on reshaping gut microecology as a target to tackle malignant disease progression. By correcting the imbalance of intestinal flora, a team from China found that the patient’s symptoms such as metabolic disorders and chronic inflammation were significantly improved, and the tendency of diabetes to worsen was blocked [51]. Actually, intestinal microecological disorders involved in CRC are also gradually known [25]. The analysis of this study showed that compared with the NC group, the abundance and diversity of flora decreased in CRC mice, which ameliorated with butyrate treatment. Microbiome sequencing analysis elaborated that *Bacteroides, Proteobacteria, Prevotellaceae, Verrucomicrobiales, Anaerostipes* and so on were obviously clustered; and *Lactobacillales* and *Enterobacterales* were also inundant at family, genus and species levels in AOM/DSS induced mice. Compared with the AOM-DSS group, butyrate not only evidently suppressed the abundance of these pernicious florae but also clearly resume the magnitude of *Actinobacteriota, Bifidobacteriales, Eubacterium, Muribaculaceae, etc*. simultaneously, which were suppressed in CRC mice.

The alteration of *Muribaculaceae*, as a beneficial flora in the gut, is very interesting in our study; and *Muribaculaceae* is gradually achievable for isolation and culture in existing papers [39]. Nevertheless, targeting *Muribaculaceae* utilized in disease is still scarce. Experts proposed that with the intestinal enrichment of *Muribaculaceae*, intestinal inflammation in elderly mice was reduced and telomerase activity was enhanced [52]. Acarbose has also been demonstrated to exert a longevity effect which is closely associated with the elevated abundance of *Muribaculaceae* in the intestinal tract [53]. Inspiringly, literature supported that *Muribaculaceae* can assist longevity mammals to release more SCFAs, and facilitate gene pool synthesis of butyrate-related pathways, which are largely suppressed in CRC [54]. Therefore, we look into the distance that *Muribaculaceae* may serve as a new orientation for future research on the microecological disturbance of CRC.

For the ecological imbalance caused by colon cancer, we should not only focus on the changes of a single immune signaling pathway or a single flora but also on the entire bacterial community that causes immunosuppression. The anticancer effect of butyrate is not simply due to its ability to regulate glycolysis metabolism of tumor cells and inflammatory cascade, but also to promote the colonization of beneficial intestinal flora [14]. Researchers have attempted to treat colon cancer by eliminating tumor-like microorganisms, transplanting antitumor fecal materials, and using phage-targeted interference to disrupt the dysfunctional microbiome; while the impact of microorganisms on the host is not single and needs to be controlled [55]. The utilization of antitumor chemoprophylaxis, such as butyrate, in combination with targeting beneficial flora colonization to improve the occurrence and progression of CRC may be worth to further exploration.

To sum up, butyrate inhibits intestinal inflammation and injury, reduces harmful and saves beneficial microbiota and reconstructs the destroyed intestinal microecology, so as to alleviate AOM/DSS induced cancer.

### Conclusion

This study identified the alleviating effect of butyrate on CRC, which involves repairing intestinal inflammation, reducing the invasion of harmful flora and reestablishing a harmonious and orderly intestinal microecology. These data provide novel and effective evidence for oral butyrate to regulate the composition of intestinal flora, so as to relieve CRC clinically. However, due to the limited sample size of animal experiments and the lack of in-depth study of molecular research, the specific mechanism of butyrate regulating intestinal microecosystem to alleviate CRC needs to be further explored.

## Acknowledgements

This work is supported by the research grants from the Science and Technology Development Project of Yancheng, China (YK2019108), the Nantong University Clinical Medicine Special Project, China (2019JZ011) and Cultivation Fund of Yancheng First People’s Hospital, China (QN2020008).

### Conflicts of interest

There are no conflicts of interest.
